# Seasonal succession of bacterial communities in cultured *Caulerpa lentillifera* detected by high-throughput sequencing

**DOI:** 10.1515/biol-2022-0001

**Published:** 2022-01-28

**Authors:** Meixia Pang, Zhili Huang, Le Lv, Xiaodong Li, Gang Jin

**Affiliations:** Postdoctoral Innovation Practice Base, Shenzhen Polytechnic, Shenzhen 518055, China; School of Applied Chemistry and Biological Technology, Shenzhen Polytechnic, Shenzhen 518055, China

**Keywords:** 16S rDNA, bacterial diversity, *Caulerpa lentillifera*, washing with tap water

## Abstract

An increasing number of microorganisms are being identified as pathogens for diseases in macroalgae, but the species composition of bacteria related to *Caulerpa lentillifera*, fresh edible green macroalgae worldwide, remains largely unclear. The bacterial communities associated with *C. lentillifera* were investigated by high-throughput 16S rDNA sequencing, and the bacterial diversities in washed and control groups were compared in this study. A total of 4,388 operational taxonomic units were obtained from all the samples, and the predominant prokaryotic phyla were *Proteobacteria*, *Bacteroidetes*, *Planctomycetes*, *Cyanobacteria*, *Actinobacteria*, *Verrucomicrobia*, *Chloroflexi*, and *Acidobacteria* in *C. lentillifera*. The bacterial diversity changed with seasons and showed an increasing trend of diversity with the rising temperature in *C. lentillifera*. There were slight reductions in the abundance and diversity of bacteria after washing with tap water for 2 h, indicating that only parts of the bacterial groups could be washed out, and hidden dangers in *C. lentillifera* still exist. Although the reduction in the abundance of some bacteria revealed a positive significance of washing *C. lentillifera* with tap water on food safety, more effective cleaning methods still need to be explored.

## 1 Introduction

The genus *Caulerpa* (*Bryopsidales*, *Chlorophyta*), a siphonous green macroalgae, is widely distributed in subtropics and tropics, such as China, Singapore, Indonesia, the Philippines, Malaysia, Vietnam, and Japan. Some species of the genus are consumed as vegetables blended with onion, vinegar, or tomatoes [1]. For example, Indonesian *Caulerpa* usually is served as a side dish in South Sulawesi [2] and is used in fresh salads in Japan and many Asian regions [3]. *C. lentillifera* (sea grape), one of the most popular edible green macroalgae [1], which looks like grapes and is thus called green caviar [4,5,6].

The algae of genus *Caulerpa* are high in several vitamins and minerals, including iron, calcium, magnesium, and iodine [7,8,9]. Moreover, *C. lentillifera* contains a high level of polyunsaturated fatty acids and multiple essential amino acids (EAA) with low-level total lipid content [10]. The EAA composition of *C. lentillifera* approaches the ideal model recommended by the Food and Agriculture Organization/World Health Organization [11], and the reported protein content varies from 3.6 to 19.4% dry weight mass of *C. lentillifera* [6,12,13]. *C. lentillifera* also has potential functions, such as antidiabetic activity [14,15], anti-inflammatory activity [16], immunostimulatory activity [17], preventing hypertension [18], as well as anticoagulant and anticancer activity [19]. In recent years, *C. lentillifera* was introduced into China for a large-scale artificial cultivation as functional seafood. *C. lentillifera* was cultured with sand-filtered seawater on a double-layer net, under which a layer of sand was laid as an attachment base for rhizomes. The artificially cultivated *C. lentillifera* were raised in muddy ponds following standard culture conditions (20.0–32.0°C, 5,000–10,000 Lux) and were harvested regularly.

Diseases caused by seafood pose a critical hazard to public health worldwide [20]. The global consumption of seafood per capita has increased over the last few years [21]. The import and domestic aquaculture of seafood have also increased. Besides, some recent human gastroenteritis outbreaks have been associated with contaminated seafood consumption [22]. More details on pathogen virulence and pathogenicity should be obtained to investigate the seafood-borne disease caused by pathogens such as norovirus and *Vibrio* [3]. There are many foodborne pathogens in the marine environments, which may attach to the surface of seafood and enter the human body on consumption in fresh and live forms, thereby leading to several health risks. For example, *Vibrio parahaemolyticus* is a facultative, anaerobic, gram-negative bacterium with a curved rod shape, usually found in an estuary or marine environment, and causes spoilage of *C. lentillifera* [3].

An increasing number of microbes are being identified as pathogens of macroalgal disease [23], but bacteria attached to *C. lentillifera* remain largely unclear for the species composition. In addition, the consumption of the cultured *C. lentillifera* directly after washing with tap water is quite common [24]. Our study aims to identify bacterial communities associated with *C. lentillifera* by high-throughput 16S rDNA sequencing and explore whether washing with tap water can eliminate some pathogenic bacteria. Our results regarding the bacterial characteristics illustrated the structure of *C. lentillifera* microflora and determined the effectiveness of washing for food safety of *C. lentillifera.*

## 2 Materials and methods

### 2.1 Sample collection and DNA extraction

*C. lentillifera* samples, cultured with sand-filtered seawater pumped from the South China Sea, were collected from culture ponds in Shenzhen, Guangdong province of China (114°03′ E/22°44′ N). *C. lentillifera* materials were collected monthly from June 2018 to May 2019, except in January as the species was absent. Then, *C. lentillifera* materials collected each time were assigned to the control group (marked as S) and washed group (marked as SW). For the washed group, *C. lentillifera* materials were soaked with chlorinated tap water for 2 h, and the water was changed four times for 30 min each during the washing process. All the samples were stored at −80.0°C until further processing. *C. lentillifera* samples were named the group marker plus the collection time. For example, S1806 was collected in June 2018 and divided into the control group; and SW1905 was collected in May 2019 and divided into the washed group (Table 1).

**Table 1:** Detail information about all samples used in this study

Samples	Group name	Subgroup name	Tag number		OTU number	Sample time	Water temperature (°C)
S1806	S	S1	33,742	1,133		June 2018	28.5
S1807	S	S1	34,397	1,733		July 2018	29.5
S1808	S	S1	40,463	1,433		August 2018	29.0
S1809	S	S2	42,789	1,223		September 2018	26.0
S1810	S	S2	31,559	1,141		October 2018	24.5
S1811	S	S2	40,601	1,222		November 2018	21.5
S1812	S	S3	41,679	1,160		December 2018	20.5
S1902	S	S3	40,713	1,129		February 2019	20.0
S1903	S	S4	42,186	719		March 2019	23.0
S1904	S	S4	40,097	1,696		April 2019	25.5
S1905	S	S4	40,377	1,358		May 2019	27.0
SW1806	SW	SW1	35,689	1,071		June 2018	28.5
SW1807	SW	SW1	31,833	1,341		July 2018	29.5
SW1808	SW	SW1	41,677	1,260		August 2018	29.0
SW1809	SW	SW2	42,573	1,259		September 2018	26.0
SW1810	SW	SW2	33,145	1,363		October 2018	24.5
SW1811	SW	SW2	42,496	510		November 2018	21.5
SW1812	SW	SW3	35,814	1,474		December 2018	20.5
SW1902	SW	SW3	45,465	701		February 2019	20.0
SW1903	SW	SW4	42,498	820		March 2019	23.0
SW1904	SW	SW4	40,855	1,507		April 2019	25.5
SW1905	SW	SW4	43,602	592		May 2019	27.0

S: control group and SW: washed group. S1, S2, S3, and S4: control *C. lentillifera* samples collected in summer, autumn, winter, and spring; and SW1, SW2, SW3, and SW4: washed *C. lentillifera* samples collected in summer, autumn, winter, and spring. All samples were collected monthly. Three replicates per season were performed.

The sampling temperature of seawater was 20.0–29.5°C with an average temperature of 25.0°C. When dividing the sampling time into four seasons, the average temperatures in summer (labeled as 1), autumn (labeled as 2), winter (labeled as 3), and spring (labeled as 4) were 29.0, 24.0, 20.25, and 25.2°C, respectively. To understand the diversities and variability of microorganisms with the change of seasons in *C. lentillifera*, all the samples were assigned to seasonal subgroups. Detailed information is shown in Table 1. All samples were collected monthly. Three replicates per season were performed.

For DNA extraction from *C. lentillifera*, TIANamp Stool DNA Kit (Tiangen, Beijing, China) was used according to the manufacturer’s protocol. The obtained DNA integrity was tested by 1% agarose gel electrophoresis and quantified using the PicoGreen dsDNA quantitation assay (Invitrogen, Carlsbad, CA), and the extracts were stored at −20.0°C.

### 2.2 16S rDNA library generation and microbiome sequencing

The universal primers 515F (5′-GTGCCAGCMGCCGCGGTAA-3′), together with 806R (5′-GGACTACHVGGGTWTCTAAT-3′), were used to amplify the bacterial 16S rRNA gene V4 hypervariable region of DNA samples following specific procedures. The polymerase chain reaction (PCR) reaction system consisted of 1× Hi-Fidelity buffer, 30 ng qualified genomic DNA, dNTP PurePeak DNA polymerase mix (200 µM, Pierce Nucleic Acid Technologies, Milwaukee, WI, USA), Platinum Taq High Fidelity Polymerase (1 unit, Life Technologies, Carlsbad, CA, USA), MgCl_2_ (2.0 mM), 0.06% BSA, along with forward and reverse primers (0.2 µM each). PCR amplification parameters were set as follows: 3 min of initial denaturation under 98.0°C; 45 s under 98.0°C, 45 s under 55.0°C, and 45 s under 72.0°C for 30 cycles; 7 min of extension under 72.0°C. Then, the Agencourt AMPure XP magnetic beads were utilized to purify the amplified PCR products, eventually dissolved into the elution buffer. The Agilent 2100 Bioanalyzer (Agilent Technologies, Santa Clara, CA, USA) was adopted to test the DNA libraries, while the HiSeq platform was used for pair-end sequencing, with the PE250 sequencing strategy was used (PE251 + 8 + 8 + 251; HiSeq SBS Kit V2, Illumina) under specific protocols.

### 2.3 Sequence analysis and bioinformatics

Clean data were obtained by filtering low-quality sequences from Raw fastq files using the program Quantitative Insights Into Microbial Ecology (version 1.9.1) (http://www.wernerlab.org/software/macqiime) [25], as described in an earlier study [26]. Then, Fast Length Adjustment of Short reads software (v1.2.11) [27] was used to merge those pair-end reads for obtaining tags that contained the V4 hypervariable region, the minimal matching length was 15 bp, whereas the mismatch ratio was 0.1 within the overlapped regions. UPARSE [28] was utilized to cluster the operational taxonomic units (OTUs) with a similarity threshold of 97%, whereas UCHIME [29] was used to identify and remove the chimeric sequences. The sequence NCBI number was PRJNA658212. For assigning OTUs to the nearest matching described taxon, the Greengenes taxonomy database (version 13_5) was adopted to query sequences for 16S rRNA genes [30]. Finally, each quality-filtered read was mapped by the usearch_global algorithm to the eventual set, which represented OTU sequences [31], to obtain the community composition of each sample.

In the samples, the microorganism alpha-diversity indices were evaluated according to the annotated data, including the observed species index, Chao I richness, ace index, Shannon index, and good coverage [32]. Among them, the observed species, Chao I richness, and ace index reflected the species richness of the bacterial community. The rarecurve function was used to calculate and plot the rarefaction curves [33], corresponding to the observed species in the R package vegan. Shannon index presents the diversity of microbial species and can be impacted by species evenness and richness of a sample community, while good coverage is a value representing sequencing coverage of the sample library. The relative abundance (RA) of the bacterial community composition of the samples was evaluated at the levels of phylum, class, and genus. Multiple comparisons of the bacterial alpha-diversity indices and RA between the different groups (or subgroups) were subject to one-way analysis of variance and Tukey’s HSD *post hoc* test using the SPSS 19.0 software. The results were presented as mean ± standard error, and differences were considered significant at *p* < 0.05.

Linear discriminant analysis effect size (LEfSe) [34] has been developed as an approach to discover and explain biomarkers for high-dimensional data. In LEfSe, statistical significance is applied in combination with the estimation of effective size and biological consistency. In this study, LEfSe was adopted to discover biomarkers based on microorganisms. In contrast, LEfSe analysis-derived linear discriminant analysis (LDA) scores were adopted for displaying the association across taxa by the cladogram (circular hierarchical tree) regarding those remarkably upregulated and downregulated microbial taxa between two groups. In each sample, the biomarker taxon RA was presented in straight dotted lines, and the medians and averages for subgroups were also plotted. The levels of the branch graph represent the phylum, class, order, family, and genus from the inner to the outer circles. The color codes and the letters indicate the groups and the taxa, respectively, that contribute to the uniqueness of the corresponding groups when LDA >2.0.

## 3 Results

### 3.1 Richness and diversity

**Figure 1:**
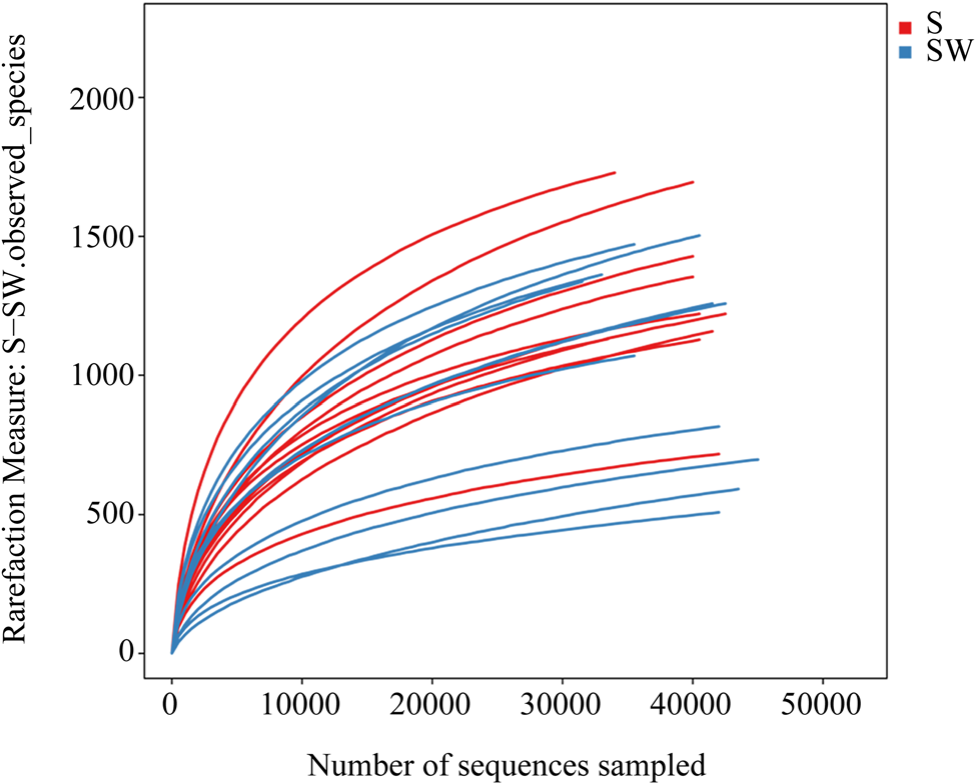
Observed species rarefaction curves of all samples. S: control group and SW: washed group.

In this study, we first analyzed the overall microbial diversity in a total of 22 *C. lentillifera* samples across four seasons. The samples were sequenced, yielding 0.86 million short-read V4 16S rRNA gene sequences (31,559–45,465 per library; Table 1). After strict quality and size filtering, high-quality sequences were clustered into 4,388 OTUs corresponding to the bacterial community (510–1,733 per sample; Table 1). Observed species rarefaction curves reached coverages of more than 0.95 (Table 2), suggesting that a very reasonable sequencing depth has been attained (Figure 1).

**Table 2:** Average alpha-diversity indices of the different group samples

Group	Subgroup	Observed species		Chao1 richness	Ace index	Shannon index	Good coverage
S	S1	1062.5 ± 239.6^a^		1453.3 ± 273.2^a^	1555.8 ± 257.5^a^	4.8 ± 0.6^a^	0.950 ± 0.009^a^
	S2	867.2 ± 60.2^a^		1243.1 ± 20.4^a^	1365.4 ± 88.2^a^	3.1 ± 0.4^b^	0.958 ± 0.006^a^
	S3	820.8 ± 17.3^a^		1213.1 ± 86.8^a^	1370.4 ± 206.1^a^	3.0 ± 0.5^b^	0.962 ± 0.000^a^
	S4	910.7 ± 353.6^a^		1291.3 ± 494.3^a^	1448.2 ± 560.8^a^	3.5 ± 0.7^ab^	0.959 ± 0.013^a^
	Total	923.9 ± 214.8		1308.1 ± 272.3	1440.8 ± 297.8	3.7 ± 0.9	0.957 ± 0.009
SW	SW1	889.8 ± 82.0^a^		1274.3 ± 162.9^a^	1399.4 ± 201.5^a^	4.2 ± 05^a^	0.955 ± 0.008^a^
	SW2	759.2 ± 349.1^a^		1104.3 ± 451.8^a^	1215.7 ± 455.7^a^	3.6 ± 1.4^a^	0.962 ± 0.014^a^
	SW3	796.8 ± 431.9^a^		1116.3 ± 517.9^a^	1241.2 ± 442.3^a^	3.6 ± 2.1^a^	0.962 ± 0.018^a^
	SW4	685.4 ± 350.3^a^		1045.7 ± 450.6^a^	1269.5 ± 425.5^a^	2.9 ± 1.1^a^	0.967 ± 0.012^a^
	Total	781.5 ± 274.6		1136.9 ± 349.5	1285.1 ± 333.6	3.6 ± 1.2	0.961 ± 0.012

S: control group and SW: washed group. S1, S2, S3, and S4: control *C. lentillifera* samples collected in summer, autumn, winter, and spring; and SW1, SW2, SW3, and SW4: washed *C. lentillifera* samples collected in summer, autumn, winter, and spring. The results were presented as mean ± standard error, and different letters indicate that there are significant differences among different groups (seasons) (*p* < 0.05).

Among all the detected OTUs, 2,946 OTUs were shared in the S and SW groups, whereas 616 and 233 OTUs were specific in the S and SW groups, respectively (Figure S1). A total of 3,562 OTUs were found in the S group, of which 919 OTUs were shared in all seasons, whereas 411, 205, 136, and 341 OTUs were specific in summer, autumn, winter, and spring, respectively (Figure S2). Most OTUs were found in summer, followed by spring, autumn, and winter. Furthermore, a total of 3,179 OTUs was found in the SW group, with 871 OTUs shared among all the seasons, and 387, 226, 171, and 253 OTUs specific in the summer, autumn, winter, and spring, respectively, and a general lower RA than in the S group (Figure S3).

The average number of observed species, the community richness, and the community diversity in each group are shown in Table 2. As for the bacterial diversity of the S group, the average values of observed species, Chao1, ace index, and Shannon index were 923.9 ± 214.8, 1308.1 ± 272.3, 1440.8 ± 297.8, and 3.7 ± 0.9, respectively. The highest values of the richness indices and diversity indices appeared in summer, and then they decreased as the temperature decreased and, finally, increased with the arrival of spring and the rise in the temperature (Figure S4a and b). In particular, the Shannon index values in autumn and winter were significantly lower than that in summer (S1_vs_S2, *p* = 0.027; S1_vs_S3, *p* = 0.036; Figure S4b). In the samples of the SW group, the average value of observed species, Chao1, ace index, and Shannon index were 781.5 ± 274.6, 1136.9 ± 349.5, 1285.1 ± 333.6, and 3.6 ± 1.2, respectively. However, the diversity decreased compared to that in the S group, with no significant difference between the two groups (*p* > 0.05, Table 2). The bacterial diversity of the SW group showed a seasonal trend similar to that observed in the S group. The richness and diversity indices appeared high in the summer and decreased in autumn and winter, with the lowest values in the spring. No significant differences were observed among the seasons (Figure S4c and d). As for the bacterial diversity of the S and SW groups in the same season, the richness and diversity indices in the SW group showed no significant difference to that observed in the S groups (*p* > 0.05, Figure S5).

### 3.2 Prokaryotic community composition and relationships based on phylum, class, and genus levels in different groups

**Figure 2:**
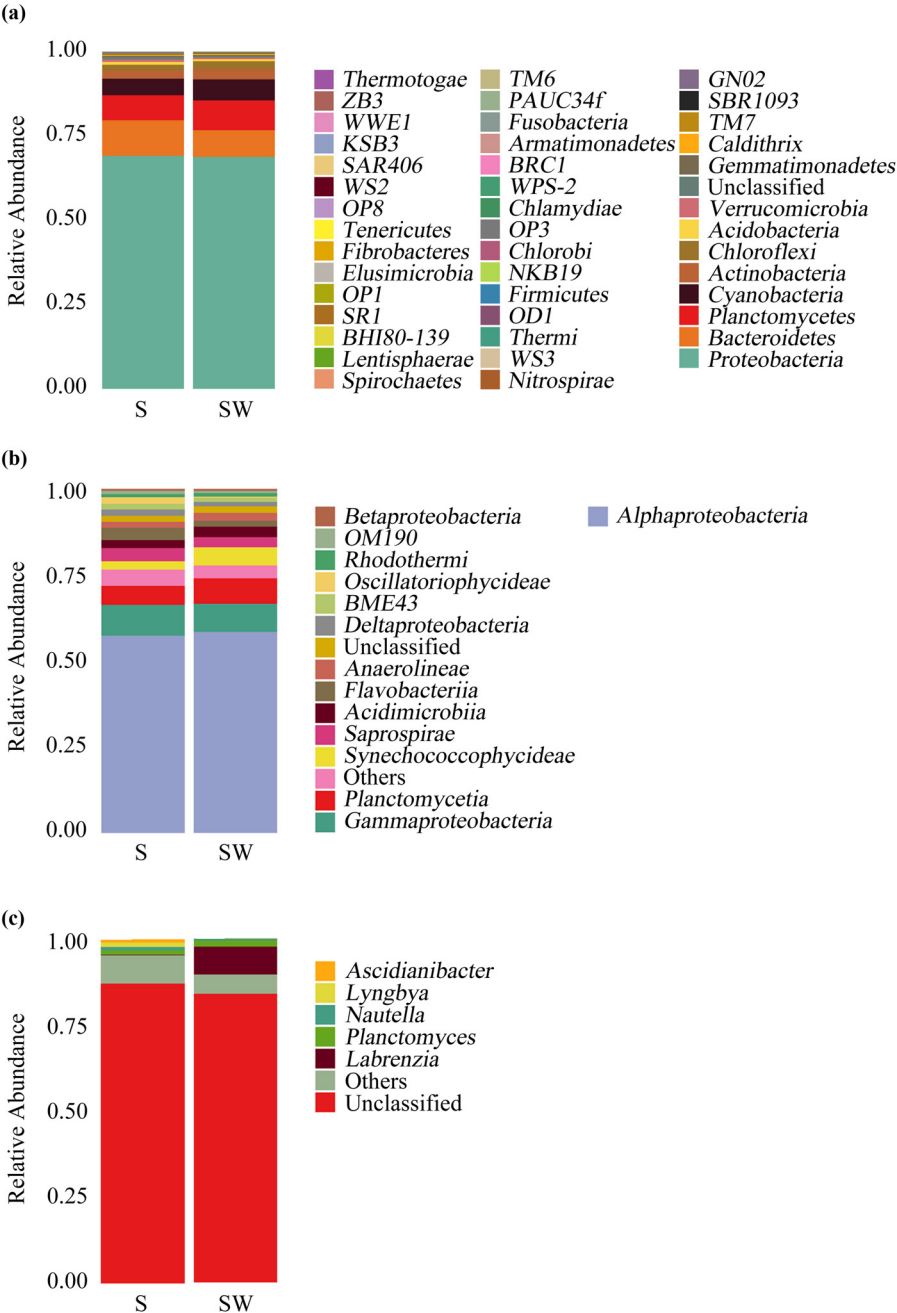
Bacterial distribution in the different groups: (a) evaluated at the phylum taxonomical level, (b) evaluated at the class taxonomical level, and (c) evaluated at the genus taxonomical level. S: control group and SW: washed group.

Forty-three prokaryotic phyla were detected in all the samples, wherein 41 and 39 were detected in the S and SW groups, respectively. The predominant phyla observed were *Proteobacteria* (68.96%), *Bacteroidetes* (9.20%), *Planctomycetes* (8.13%), *Cyanobacteria* (5.57%), *Actinobacteria* (2.72%), *Chloroflexi* (2.04%), *Acidobacteria* (0.73%), and *Verrucomicrobia* (0.59%), which accounted for more than 98% of all the sequences. The unclassified prokaryotic phyla constituted 0.58% of all the sequences (Table S1, Figure 2a). As shown in Figure 2a and Table S1, the five most abundant bacterial communities were *Proteobacteria*, *Bacteroidetes*, *Planctomycetes*, *Cyanobacteria*, and *Actinobacteria* in both S and SW groups, accounting for 69.13 and 68.80%, 10.51 and 7.88%, 7.43 and 8.83%, 4.90 and 6.25%, and 2.36 and 3.08% in S and SW groups, respectively.

In the total samples, 14 prokaryotic classes were detected in addition to others (RA less than 0.5% in all the samples) (4.22%), and the unclassified prokaryote at the class level accounted for 1.82% of all sequences (Table S1). The RAs of prokaryotes at the Class level in different groups are shown in Figure 2b. *Alphaproteobacteria* (*Proteobacteria*) and *Gammaproteobacteria* (*Proteobacteria*) were the most and the second most predominant classes, respectively. *Alphaproteobacteria* accounted for 57.34 and 58.43% of the reads, and *Gammaproteobacteria* accounted for 8.97 and 8.18% of the reads in the S and SW groups, respectively. The dominant bacterial communities in the S group also included *Planctomycetia* (*Planctomycetes*), *Saprospirae* (*Spirochaetes*), and *Flavobacteriia* (*Bacteroidetes*), accounting for 5.51, 3.84, and 3.55%, respectively (Table S1 and Figure 2b). The dominant bacterial communities in the SW group also included *Planctomycetia*, *Synechococcophycideae* (*Cyanobacteria*), and *Acidimicrobiia* (*Actinobacteria*), accounting for 7.41, 5.27, and 3.03%, respectively (Table S1 and Figure 2b).

Only five prokaryotic genera were detected in all the samples in addition to others (6.89%), and the unclassified prokaryote at the genus level accounted for 85.53% of all the sequences (Table S1). The RA of prokaryotes at the genus level in different groups is shown in Figure 2c. The S group was dominated by *Planctomyces* (*Planctomycetia*, *Planctomycetes*; 1.27%) and *Lyngbya* (*Cyanobacteria*, *Cyanophyta*; 1.25%), and the SW group was dominated by *Labrenzia* (*Alphaproteobacteria*, *Proteobacteria*; 8.11%) and *Planctomyces* (1.72%) apart from those unclassified ones (Table S1 and Figure 2c).

**Figure 3:**
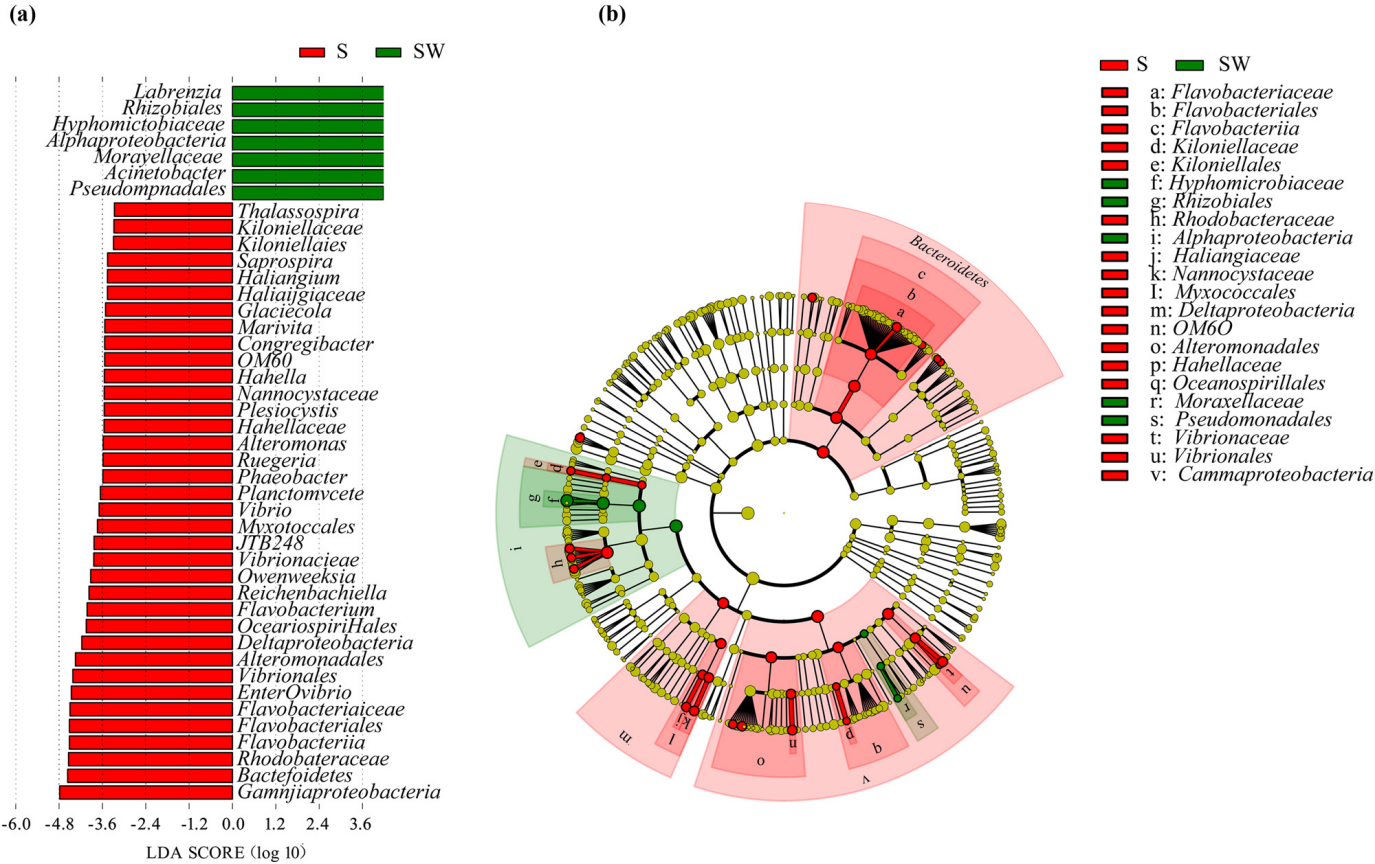
LEfSe analysis in S and SW groups: (a) LDA scores (log 10) derived from LEfSe analysis, showing the biomarker taxa for S and SW and (b) cladogram generated from LEfSe analysis showing the relationship between taxon. S: control group and SW: washed group.

For better understanding, the relationship between the bacterial community and the diverse structures of those treated *C. lentillifera*, this study conducted LEfSe analysis for determining those high-dimensional biomarker bacterial taxa of S versus SW samples (Figure 3a). Then, the cladogram (Figure 3b) was constructed to display the associations among the biomarker taxa. The results revealed that at the phylum level, *Bacteroidetes* was a biomarker bacteria related to the S group. At the class level, *Flavobacteriia*, *Deltaproteobacteria* (*Proteobacteria*), and *Gammaproteobacteria* were associated with the S group, and *Alphaproteobacteria* was associated with the SW group. The high-dimensional biomarker genera, such as *Owenweeksia* (*Flavobacteriia*, *Bacteroidetes*), *Flavobacterium* (*Flavobacteriia*, *Bacteroidetes*), *Thalassospira* (*Alphaproteobacteria*, *Proteobacteria*), *Marivita* (*Alphaproteobacteria*, *Proteobacteria*), *Ruegeria* (*Alphaproteobacteria*, *Proteobacteria*), *Haliangiaceae* (*Deltaproteobacteria*, *Proteobacteria*), *Plesiocystis* (*Deltaproteobacteria*, *Proteobacteria*), *Alteromonas* (*Gammaproteobacteria*, *Proteobacteria*), *Glaciecola* (*Gammaproteobacteria*, *Proteobacteria*), *Congregibacter* (*Gammaproteobacteria*, *Proteobacteria*), *Hahella* (*Gammaproteobacteria*, *Proteobacteria*), *Enterovibrio* (*Gammaproteobacteria*, *Proteobacteria*), and *Vibrio* (*Gammaproteobacteria*, *Proteobacteria*), were associated with S group, and genera *Labrenzia* and *Acinetobacter* (*Gammaproteobacteria*, *Proteobacteria*) were related to SW group (Figure 3). As mentioned above, only *Labrenzia* has a RA of more than 0.5% in the groups.

### 3.3 Changes in the bacterial communities in two groups across the seasons

The distribution of the dominant bacterial groups in the main phyla exhibited no changes across seasons for S and SW groups. *Proteobacteria*, *Bacteroidetes*, *Planctomycetes*, *Cyanobacteria*, and *Actinobacteria* were listed among the top five bacterial communities in different seasons (Table 3). In the S group, the abundance of *Planctomycetes* was the highest in summer, which decreased significantly in the other three seasons (S1_vs_S2, *p* = 0.007; S1_vs_S3, *p* = 0.006; and S1_vs_S4, *p* = 0.004; Figure S6a). Other dominant bacterial communities showed no significant temporal differences in the S group (*p* > 0.05, Table 3). In addition, the abundance of dominant bacterial communities showed no significant difference among these four seasons in the SW group (*p* > 0.05, Table 3).

**Table 3:** The main abundant bacterial phyla associated with the S and SW across seasons

Group	Subgroup	*Proteobacteria*	*Bacteroidetes*	*Planctomycetes*	*Cyanobacteria*	*Actinobacteria*
S	S1	52.7 ± 10.7^a^	10.9 ± 2.9^a^	14.0 ± 3.5^a^	7.0 ± 10.6^a^	5.0 ± 3.7^a^
	S2	77.6 ± 6.6^a^	8.8 ± 2.6^a^	5.9 ± 0.9^b^	2.8 ± 3.7^a^	1.3 ± 0.2^a^
	S3	77.6 ± 7.2^a^	8.2 ± 1.9^a^	4.7 ± 1.1^b^	3.6 ± 2.0^a^	1.6 ± 0.3^a^
	S4	69.2 ± 11.8^a^	13.6 ± 6.2^a^	5.1 ± 0.8^b^	6.1 ± 7.9^a^	1.7 ± 1.3^a^
SW	SW1	55.1 ± 13.0^a^	10.0 ± 3.0^a^	12.3 ± 6.9^a^	9.8 ± 15.5^a^	4.8 ± 4.5^a^
	SW2	67.9 ± 14.2^a^	10.0 ± 6.6^a^	10.5 ± 4.8^a^	2.2 ± 2.1^a^	3.9 ± 1.1^a^
	SW3	71.9 ± 24.8^a^	6.4 ± 6.0^a^	8.4 ± 5.9^a^	6.6 ± 8.3^a^	2.5 ± 0.6^a^
	SW4	75.7 ± 17.0^a^	5.8 ± 5.0^a^	5.2 ± 3.2^a^	8.4 ± 13.3^a^	1.3 ± 1.2^a^

S: control group and SW: washed group. S1, S2, S3, and S4: control *C. lentillifera* samples collected in summer, autumn, winter, and spring; and SW1, SW2, SW3, and SW4: washed *C. lentillifera* samples collected in summer, autumn, winter, and spring. The results were presented as mean ± standard error, and different letters indicate that there are significant differences among different groups (seasons) (*p* < 0.05).

The top five ranked dominant bacterial groups belonging to the main classes exhibited slight changes in the S group. The most and the second most predominant bacterial communities observed in the S group for all the seasons were *Alphaproteobacteria* (35.4–70.4%) and *Gammaproteobacteria* (5.4–11.5%), respectively (Table 4). In addition, *Planctomycetia* (9.8%), *Synechococcophycideae* (5.2%), and *Acidimicrobiia* (4.9%) were the other three dominant bacterial classes observed in summer; *Planctomycetia* (4.4 and 3.8%, respectively), *Saprospirae* (3.7 and 4.4%, respectively), and *Flavobacteriia* (2.5 and 2.0%, respectively) were dominant bacterial classes in autumn and winter. In comparison, *Flavobacteriia* (6.1%), *Saprospirae* (4.2%), and *Planctomycetia* (4.0%) were dominant bacterial classes in spring (Table 4). Among these dominant bacterial classes in the S group, the abundance of *Alphaproteobacteria* was the lowest in summer, increased significantly in autumn (*p* = 0.02) and winter (*p* = 0.022), and then decreased again with the spring coming (Table 4 and Figure S6b). Differently, *Planctomycetia* showed the highest abundance in summer, which significantly decreased in the other three seasons (S1_vs_S2, *p* = 0.019; S1_vs_S3, *p* = 0.019; and S1_vs_S4, *p* = 0.013; Table 4 and Figure S6b). Other dominant bacterial classes demonstrated no different abundances across seasons (*p* > 0.05, Table 4).

**Table 4:** The main abundant bacterial class associated with the S and SW across seasons

Group	Subgroup	*Alphaproteobacteria*	*Gammaproteobacteria*	*Planctomycetia*	*Synechococcophycideae*	*Saprospirae*	*Acidimicrobiia*	*Flavobacteriia*	*Anaerolineae*
S	S1	35.4 ± 14.4^a^	11.5 ± 3.8^a^	9.8 ± 2.9^a^	5.2 ± 8.1^a^	3.3 ± 0.5^a^	4.9 ± 3.7^a^	3.0 ± 1.9^a^	3.8 ± 1.9^a^
	S2	67.3 ± 5.2^b^	8.6 ± 2.6^a^	4.4 ± 0.5^b^	1.1 ± 1.4^a^	3.7 ± 1.5^a^	1.2 ± 0.2^a^	2.5 ± 0.4^a^	0.2 ± 0.2^b^
	S3	70.4 ± 7.7^b^	5.4 ± 0.1^a^	3.8 ± 0.4^b^	1.9 ± 0.3^a^	4.4 ± 0.5^a^	1.5 ± 0.3^a^	2.0 ± 1.4^a^	1.6 ± 1.0^ab^
	S4	57.8 ± 8.1^ab^	9.4 ± 4.6^a^	4.0 ± 0.7^b^	1.8 ± 1.6^a^	4.2 ± 2.8^a^	1.6 ± 1.3^a^	6.1 ± 5.2^a^	1.7 ± 0.5^ab^
SW	SW1	42.3 ± 17.3^a^	9.3 ± 5.1^a^	9.8 ± 6.7^a^	8.8 ± 14.3^a^	4.0 ± 1.1^a^	4.7 ± 4.4^a^	2.5 ± 0.7^a^	3.5 ± 2.4^a^
	SW2	58.4 ± 18.1^a^	7.6 ± 2.8^a^	9.1 ± 3.6^a^	1.2 ± 1.1^a^	4.8 ± 4.1^a^	3.9 ± 1.1^a^	1.3 ± 0.7^a^	2.5 ± 2.7^a^
	SW3	58.6 ± 36.1^a^	10.9 ± 9.5^a^	7.2 ± 5.2^a^	4.8 ± 5.9^a^	2.1 ± 2.3^a^	2.5 ± 0.6^a^	1.6 ± 0.7^a^	0.8 ± 0.5^a^
	SW4	67.7 ± 21.6^a^	6.7 ± 4.3^a^	4.4 ± 2.7^a^	7.8 ± 12.4^a^	1.4 ± 1.2^a^	1.2 ± 1.2^a^	1.6 ± 1.1^a^	2.1 ± 2.0^a^

S: control group and SW: washed group. S1, S2, S3, and S4: control *C. lentillifera* samples collected in summer, autumn, winter, and spring; and SW1, SW2, SW3, and SW4: washed *C. lentillifera* samples collected in summer, autumn, winter, and spring. The results were presented as mean ± standard error, and different letters indicate that there are significant differences among different groups (seasons) (*p* < 0.05).

In the SW group, the dominant bacterial groups belonging to the main classes observed in summer were *Alphaproteobacteria* (42.3%), *Planctomycetia* (9.8%), *Gammaproteobacteria* (9.3%), *Synechococcophycideae* (8.8%), and *Acidimicrobiia* (4.7%). In autumn, the dominant bacterial communities were *Alphaproteobacteria* (58.4%), *Planctomycetia* (9.1%), *Gammaproteobacteria* (7.6%), *Saprospirae* (4.8%), and *Acidimicrobiia* (3.9%). In winter, the dominant bacterial communities were *Alphaproteobacteria* (58.6%), *Gammaproteobacteria* (10.9%), *Planctomycetia* (7.2%), *Synechococcophycideae* (4.8%), and *Acidimicrobiia* (2.5%). In spring, the dominant bacterial communities were *Alphaproteobacteria* (67.7%), *Synechococcophycideae* (7.8%), *Gammaproteobacteria* (6.7%), *Planctomycetia* (4.4%), and *Anaerolineae* (2.1%) (Table 4). However, there were no significant differences in the bacterial abundance across seasons in the SW group (*p* > 0.05, Table 4).

## 4 Discussion

A macroalgal community contains bacteria, fungi, diatoms, protozoa, spores, and larvae of marine invertebrates [35]. Among these attaching organisms, many bacteria and fungi have been identified as pathogens of macroalgal diseases [23]. However, bacteria show high abundance in the primary colonizers [36], whereas fungi are relatively rare in the sea [37]. In this study, the seasonal time-series autocorrelation of bacterial diversity in *C. lentillifera* was conducted by high-throughput 16S rDNA sequencing and revealed that washing with tap water slightly alters the microbiome of *C. lentillifera*.

Overall, in the present study, 5 major genera and 14 major classes of bacteria were detected in 43 phyla in *C. lentillifera* (Table S1). *Proteobacteria* and *Bacteroidetes* constituted the most abundant bacterial phyla associated with *C. lentillifera*, which was consistent with the earlier studies on other seaweeds [38,39], such as *Laminaria saccharina* [39], *L. hyperborea* [40], *Ulva australis* [41], *C. racemosa* [42], *Cystoseira compressa* [43], and *Sargassum muticum* [44]. *Alphaproteobacteria* and *Gammaproteobacteria* were the most and the second most predominant classes related to *C. lentillifera*, accounting for an average of 57.89 and 8.58% of the reads in the S and SW groups, respectively. While at the genus level, only five main prokaryotic genera were detected in addition to others (6.89%), and the abundance of unclassified prokaryote reached to 85.53% (Table S1), which needs to be further studied.

Temporal variations of *C. lentillifera-*related bacterial microbial taxonomic composition were measured. The highest diversity of the *C. lentillifera* bacterial community was revealed in summer, followed by spring, autumn, and winter, respectively, according to OUT richness and alpha diversity (Table 2), indicating that the bacterial diversity increases with the temperature of seasons. However, no significant temporal differences were found in the bacterial structure in *C. lentillifera*, which may be due to the relatively stable temperature of Shenzhen throughout the year. Shenzhen (113°46′–114°37′ E, 22°27′–22°52′ N), one of the coastal cities in the south of China and near Hong Kong, has a mild climate with an annual average temperature of 23.0°C. The sampling temperature of seawater in this study was 20.0–29.5°C with an average temperature of 25.0°C, with a small shift across seasons. In addition, slight changes of the dominant bacterial groups contributed most of the dissimilarity across the seasons in *C. lentillifera*. The most pronounced temporal changes in the microbial community of *C. lentillifera* were abundantly increased in *Planctomycetes* in summer (Table 3), which occurred primarily due to the increase of *Planctomycetia* (Table 4). *Planctomycetes* have been recognized as capable of mineralizing organic matters into inorganic counterparts, which fulfills the nutritional demands of the macroalgae [45,46,47]. Meanwhile, macroalgae are rich in *Planctomycetes* [47,48], the RA of which varies greatly depending on seaweed species and seasons [35]. The summer increase of *Planctomycetes* in the present study is congruent with the studies reported on *L. hyperborean* [40] and *Sargassum muticum* [44].

To our knowledge, this is the first study to investigate whether washing with tap water alters the microbiome associated with *C. lentillifera* using high-throughput 16S rRNA gene sequencing and modern multivariate data analyzing software programs. The LEfSe analysis and cladogram visualization (Figure 3) revealed few types of biomarker bacteria associated with washed *C. lentillifera*, and the most representative one was *Labrenzia*. The RA of the genus *Labrenzia*, belonging to the family *Rhodobacteraceae*, was 50 times higher in *C. lentillifera* after washing. *Labrenzia* is the aerobic anoxygenic phototrophic bacterium that can generate little bacteriochlorophyll [49]. The abundance of *Labrenzia* was found to be higher in healthy *C. lentillifera* as compared to the diseased samples, which may contribute to the photosynthesis of algae [50]. Because the genus *Labrenzia* was difficult to elute by tap water, it resulted in an increased abundance in *C. lentillifera* even after washing, suggesting that there might be a symbiotic relationship between *C. lentillifera* and *Labrenzia.*

In China, the consumption of *C. lentillifera* directly after washing is quite common, which might lead to be bacterial infection. More types of high-dimensional biomarkers bacteria at different levels were associated with the S group, such as phylum of *Bacteroidetes*, classes of *Flavobacteriia*, *Flavobacterium*, *Deltaproteobacteria*, and *Gammaproteobacteria*, and genera of *Haliangiaceae*, *Plesiocystis*, *Alteromonas*, *Glaciecola*, or *Congregibacter* (Figure 3). The abundance of these bacteria significantly decreased in *C. lentillifera* after washing, indicating that the bacterial groups were on the surface of *C. lentillifera* and relatively easy to elute by tap water. In the current study, *Vibrio* was one of the representatives of high-dimensional biomarker genera associated with the S group. It significantly decreased abundance in *C. lentillifera* after washing, which benefits consumers’ health, as it could cause seafood-borne diseases (Figure 3). For example, *Vibrio cholerae* is the pathogen causing human cholera. These ancient and widespread infectious diseases have caused many epidemics worldwide, mainly manifested as severe vomiting, diarrhea, water loss, and high mortality, and are considered an international quarantine infectious disease [51]. *V. parahaemolyticus* is another species belonging to the *Vibrio* genera. Eating food containing these bacteria can cause food poisoning, also known as halophilic bacteria food poisoning, the main clinical symptoms of which are acute onset, abdominal pain, vomiting, diarrhea, and watery stool [52].

Although LEfSe analysis revealed that the abundances of some bacteria groups associated with *C. lentillifera* were significantly decreased after washing with tap water, there was only a marginal reduction in both richness and diversity of the entire bacterial communities according to the results of α-diversity (Table 2). It was notable that there were 233 OTUs specific for the SW group (Figure S1), which may come from the tap water microbiome used for washing, or it may be caused by individual differences. The reduction in the abundance of harmful bacteria (such as *Vibrio*) in this study showed that washing *C. lentillifera* with tap water had a certain positive significance for food safety. In addition, there were still relatively abundant bacterial communities in *C. lentillifera* after washing with tap water, which may have hidden dangers to food safety, and more effective cleaning methods need to be explored. However, the results obtained by high-throughput sequencing in this study should be validated by specific and dedicated assays (microbial culture techniques or PCR), and specific norms for microbial food safety tasks need to be applied in the future.

## 5 Conclusion

In this study, a total of 4,388 OTUs were obtained from all the samples, and 5 major genera and 14 major classes of bacteria were detected in 43 phyla in *C. lentillifera*. The predominant prokaryotic phyla were *Proteobacteria*, *Bacteroidetes*, *Planctomycetes*, *Cyanobacteria*, *Actinobacteria*, *Verrucomicrobia*, *Chloroflexi*, and *Acidobacteria*. We demonstrated that the bacterial diversities associated with *C. lentillifera* increase with the temperature of seasons, with no significant temporal shifts. Slight changes in the dominant bacterial groups contributed most of the dissimilarity in bacterial communities across the seasons in *C. lentillifera*. For instance, the abundance of *Planctomycetes* in *C. lentillifera* was significantly increased in summer than in the other three seasons, which occurred mostly due to the increase of *Planctomycetia*. The increased abundance of *Labrenzia* in washed *C. lentillifera* suggested that there was a symbiotic relationship between *C. lentillifera* and *Labrenzia.* In contrast, the significant reduction in the abundance of harmful bacteria (such as *Vibrio*) showed that washing *C. lentillifera* with tap water is beneficial for human health. However, we found that both the richness and diversity of the bacterial communities associated with *C. lentillifera* only slightly decreased after washing with tap water, indicating hidden dangers in *C. lentillifera* for food safety, and more effective cleaning methods need to be explored.
